# ANXA6 Contributes to Radioresistance by Promoting Autophagy via Inhibiting the PI3K/AKT/mTOR Signaling Pathway in Nasopharyngeal Carcinoma

**DOI:** 10.3389/fcell.2020.00232

**Published:** 2020-04-16

**Authors:** Qianping Chen, Wang Zheng, Lin Zhu, Dan Yao, Chen Wang, Yimeng Song, Songling Hu, Hongxia Liu, Yang Bai, Yan Pan, Jianghong Zhang, Jian Guan, Chunlin Shao

**Affiliations:** ^1^Institute of Radiation Medicine, Shanghai Medical College, Fudan University, Shanghai, China; ^2^Department of Radiation Oncology, Nanfang Hospital, Southern Medical University, Guangzhou, China

**Keywords:** ANXA6, radioresistance, autophagy, PI3K/AKT/mTOR, NPC

## Abstract

Radiotherapy is a conventional and effective treatment method for nasopharyngeal carcinoma (NPC), although it can fail, mainly because radioresistance results in residual or recurrent tumors. However, the mechanisms and predictive markers of NPC radioresistance are still obscure. In this study, we identified Annexin A6 (ANXA6) as a candidate radioresistance marker by using Tandem Mass Tag quantitative proteomic analysis of NPC cells and gene chip analysis of NPC clinical samples with different radiosensitivities. It was observed that a high expression level of ANXA6 was positively correlated with radioresistance of NPC and that inhibition of ANXA6 by siRNA increased the radiosensitivity. The incidence of autophagy was enhanced in the established radioresistant NPC cells in comparison with their parent cells, and silencing autophagy with *LC3* siRNA (siLC3) sensitized NPC cells to irradiation. Furthermore, *ANXA6* siRNA (siANXA6) suppressed cellular autophagy by activating the PI3K/AKT/mTOR pathway, ultimately leading to radiosensitization. The combination of siANXA6 and CAL101 (an inhibitor of PI3K, p-AKT, and mTOR, concurrently) significantly reversed the above siANAX6-reduced autophagy. Suppression of PI3K/AKT/mTOR by CAL101 also increased the expression of ANXA6 in a negative feedback process. In conclusion, this study revealed for the first time that ANXA6 could promote autophagy by inhibiting the PI3K/AKT/mTOR pathway and that it thus contributes to radioresistance of NPC. The significance of this is that ANXA6 could be applied as a new predictive biomarker of NPC prognosis after radiotherapy.

## Introduction

Nasopharyngeal carcinoma (NPC) is a cancer arising from nasopharynx epithelium. Compared with other types of tumors, NPC has a low incidence rate and a unique geographic distribution pattern. In 2018, there were about 129,000 new cases of NPC in the world, according to the International Agency for Research on Cancer (Chen et al., 2019). Nevertheless, the geographical distribution of NPC patients across the globe is extremely unbalanced, with more than 70% of new cases located in eastern and southeastern Asia, especially in southern China (Ferlay et al., 2015; Chen et al., 2019). Radiotherapy is the primary and only curative treatment for NPC because of the special anatomical location and high sensitivity to radiation of this cancer. Recently, with the development of intensity-modulated radiotherapy (IMRT), radiotherapy has achieved 5-year overall survivals of 90 and 84% for stage I and stage IIA NPC, respectively (Lee et al., 2005). However, some advanced patients still exhibit radioresistance, leading to the failure of radiotherapy (Li et al., 2006; Liu et al., 2013). At present, few biomarkers have been used in the clinic to predict the radioresistance of NPC (Chen and Hu, 2015).

ANXA6 (Annexin A6) belongs to the highly conserved annexin protein family and has been implicated in mediating the endosome aggregation and vesicle fusion in secreting epithelia during exocytosis (Enrich et al., 2011). Like other annexins, ANXA6 binds to phospholipids and functions in a Ca^2+^-dependent manner, thus activating cellular membrane in a dynamic, reversible, and regulated way (Grewal et al., 2010). Upon cell activation, ANXA6 is recruited to the plasma membrane, endosomes, and caveolae/membrane rafts to interact with signaling proteins to handle intracellular Ca^2+^ signaling (Enrich et al., 2011) and inhibits the epidermal growth factor receptor (EGFR) and Ras signaling pathway (Grewal et al., 2010; Koese et al., 2013). Overexpression of ANXA6 has been reported to be associated with poor prognosis of tumors (Lomnytska et al., 2011). Moreover, there is evidence that the expression of ANXA6 might represent new Ca^2+^ effectors that regulate converging steps of autophagy in hepatocytes (Enrich et al., 2017). However, its role in radiosensitivity remains unknown.

When exposed to adverse environmental conditions, cells degrade their own content to recycle cellular building blocks through a process of autophagy (Katheder and Rusten, 2017). A large body of literature have connected autophagy to cancer, and some studies have focused on the function of autophagy in radioresistance. Autophagy is also called programed cell death type II, which is different from the apoptotic type I death pathway (Choi et al., 2013). It is a highly conserved catabolic process that maintains cellular homeostasis by targeting damaged proteins or organelles to lysosomal compartments for degradation (Zhang et al., 2019). Autophagy has potential roles in radioresistance of cancer cells such as colorectal cancer, breast cancer, glioma, and pancreatic cancer (Yao et al., 2003; Chaachouay et al., 2011, 2015; Wang et al., 2013; Koukourakis et al., 2016). Inhibition of the PI3K/AKT/mTOR signaling pathway is one of the classical pathways for autophagy induction. There is increasing evidence showing that the activation of autophagy associated with the inhibition of the PI3K/AKT/mTOR pathway regulates many biological processes, including platelet activation, psoriasis regulation, and osteoarthritis attenuation (Varshney and Saini, 2018; Wang et al., 2018; Cai et al., 2019). However, few studies have focused on the role of autophagy induced by PI3K/AKT/mTOR inhibition in the radioresistance of NPC.

This study found that the level of autophagy increased along with the enhancement of radioresistance of NPC cells and that ANXA6 was highly expressed in both radioresistant NPC cells and NPC patients. The relationship among ANXA6, autophagy, and the PI3K/AKT/mTOR pathway in the induction of radioresistance of NPC was further investigated.

## Materials and Methods

### Cell Culture and Irradiation

Nasopharyngeal carcinoma cell lines of CNE1 and HNE2 were purchased from Shanghai Cell Bank in 2016. The cells were used up to a passage number of 15. The cells were cultured with RPMI-1640 medium (Gibco, Hangzhou, China) supplied with 10% fetal bovine serum (Gibco Invitrogen, Grand Island, NY, United States), 100 U/ml penicillin, and 100 mg/ml streptomycin and maintained at 37°C in an atmosphere of 5% CO_2_. Testing for mycoplasma was performed on a monthly basis.

To generate a radioresistant cell line, CNE1 cells were irradiated with fractionated doses of 2, 2, 4, 4, 4, 4, 6, 6, 6, 6, 8, and 8 Gy (60 Gy in total) of γ-rays (^137^Cs, Gammacell-40, MDS Nordion, Canada) at a dose rate of 0.73 Gy/min. Cells were irradiated with 2 Gy once a day, 4 Gy once a week, 6 Gy every 10 days, and 8 Gy every 2 weeks. One day before irradiation, 1.5 × 10^6^ cells were seeded in a 60 mm culture dish. After each irradiation, the cells were passaged two or more times so that they had enough vitality for the next irradiation. After fractionated irradiation of 60 Gy, the surviving cells, named CNE1R cells, became more radioresistant than CNE1 cells.

### Colony Formation Assay

The radiosensitivities of CNE1, CNE1R, and HNE2 cells were assessed by cell colony-formation assay. Cells were plated in the six-well plate at a density of 150, 300, 800, and 1600 cells/well. After full attachment, they were exposed to 0, 2, 4, and 6 Gy, respectively. At 8–12 days after radiation, cell colonies were fixed with methanol for 20 min and stained with 0.1% crystal violet for 30 min in order to count them. The cell survival curve was fitted using the single-hit multitarget model.

### Western Blot Assay

Total cellular proteins were extracted using SDS lysis buffer (250 nM Tris–HCL, pH 7.4, 2.5% SDS) with 100 mM phenylmethanesulfonyl fluoride (PMSF) (Beyotime, Biotechnology, Haimen, China). After denaturing at 100°C for 10 min, aliquots of protein (20 μg/sample) were electrophoresed on 10 or 12% polyacrylamide gel (according to the molecular weight of goal proteins) using an electrophoresis system (Bio-Rad Laboratories Inc., CA, United States). After electrophoresis, proteins were transferred to a PVDF membrane, blocked with 5% skim milk in Tris-buffered saline/Tween 0.05% (TBST) for 2 h and then incubated overnight at 4°C with a primary antibody of anti-ANXA6 antibody (1:2000, Abclonal), anti-P62 (1:1000, Cell signaling Technology), anti-LC3 (1:1000, Cell signaling Technology) or anti-Actin (1:20000, Abclonal). Then the membrane was triply washed with TBST at room temperature for 10 min and labeled with a peroxidase-conjugated secondary antibody (1:5000, Beyotime Biotechnology) for 2 h. Proteins in the membrane were detected by the enhanced chemiluminescence system (ECL kit, Millipore, St. Louis, MO, United States), and band images were analyzed with the Bio-Rad ChemiDoc XRS system.

### Tandem Mass Tag (TMT) Quantitative Proteomic Analysis

Each protein sample in lysis buffer (8 M urea, 1% Protease Inhibitor Cocktail) was sonicated triply using a high-intensity ultrasonic processor (Scientz, Ningbo, China). The remaining debris was removed by centrifugation at 12,000 g at 4°C for 10 min. Finally, the supernatant was collected, and the protein concentration was determined with a BCA kit according to the manufacturer’s instruction. Trypsin was then used for digestion to generate peptide. The peptide was desalted by Strata X C18 SPE column (Phenomenex, CA, United States) and vacuum dried. The tryptic peptides were digested into fractions and separated by high pH reverse-phase HPLC using Agilent 300Extend C18 column (5 μm particles, 4.6 mm ID, 250 mm length) and subjected to a NSI source followed by tandem mass spectrometry (MS/MS) in a Q ExactiveTM Plus (Thermo, MA, United States) coupled online to the UPLC. The MS/MS data were processed with the Maxquant search engine (v.1.5.2.8).

### Patients and Clinical Gene Chip

This study was carried out in accordance with the recommendations of international guidelines and ethical standards. All subjects gave written informed consent in accordance with the Declaration of Helsinki. For the experiments using human participants or data, prior approval was obtained from the Nanfang Hospital of Southern Medical University Institutional Board (Guangzhou, China).

A total of 185 NPC patients were recruited in this study, comprising 124 radiosensitive (87 male, 37 female, mean age 46 years) and 61 radioresistant patients (49 male, 12 female, mean age 46 years). All patients accepted a standard regimen of radiotherapy. Three months after therapy was completed, a contrast-enhanced Magnetic Resonance Imaging (MRI) or Computed Tomography (CT) scan and a thorough examination were performed to evaluate short-term efficacy. According to the Response Evaluation Criteria in Solid Tumor (RECIST) guideline, those with complete response (CR) and partial response (PR) were classified into the radiosensitive group, while those with stable disease (SD) and progressive disease (PD) were classified into the radioresistant group. The clinicopathologic characteristics of NPC tissues used in the present study are demonstrated in Supplementary Table S1.

Three patients in the radioresistant group were selected to provide paraffin samples for gene chip detection. Paraffin samples were collected before and after treatment and divided into these two groups for differential gene study. Paraffin samples were then extracted using the RNeasy kit (Tiangen, Beijing, China), and gene chips were obtained by hybridization, washing, and staining. We analyzed gene chips by the standardized method provided by the Affymetrix human U133 + 2.0 chip (Bohao Biotechnology Co., Shanghai, China).

### RNA Extraction and Quantitative Real-Time PCR Assay

Total RNA was extracted from CNE1, CNE1-R, and HNE2 cells for RT-PCR using total RNA Kit I (Omega, Norcross, GA, United States). Reverse transcription of total RNA to cDNA was carried out in 20 μl reaction reagents of the qRT-PCR Kit (Tiangen, Beijing, China) according to the manufacturer’s protocol. For *ANXA6* gene, the forward primer was 5′-ACG GTT GAT TGT GGG CCTG-3′) and the reverse primer was 5′- GTG CAT CTG CTC ATT GGT CC-3′. For β*-actin* gene, the forward primer was 5′-CAT GTA CGT TGC TAT CCA GGC-3′ and the reverse primer was 5′- CTC CTT AAT GTC ACG CAC GAT-3′. The optimal PCR amplification procedure was performed for 40 cycles with pre-denaturation at 95°C for 15 min, denaturation at 95°C for 10 s, and annealing and extension at 60°C for 32 s.

### siRNA Transfection

CNE1 and CNE1-R cells were transferred with *ANXA6* siRNA (siANXA6) (target sequence: CGG GCA CTT CTG CCA AGA AAT), *LC3* siRNA (siLC3) (target sequence: GAG UGA GAA AGA UGA AGA UTT), and siRNA negative control of random sequence using riboFECTTM CP Transfection Agent (Ribobio, Guangzhou, China). The transfection efficiency was evaluated by PCR at 24–72 h after transfection, and the survival of siLC3 transfected cells was measured with a colony formation assay.

### Autophagy Flux Assay

Cells were plated at a density of 2 × 10^5^ per well and allowed to adhere overnight. Cells in about 70% confluence were transfected with *mRFP-GFP-LC3* double-labeled adenovirus (Ad-*mRFP-GFP-LC3*) to label autophagosome (Hanbio Biotechnology Co., Shanghai, China) according to the manufacturer’s instruction. After 2 h of transfection, the cells were cultured in fresh medium for 48 h then washed with pre-cooled PBS twice and stained with DAPI. The intracellular autophagy was observed by a high-content imaging system (ImageXpress Micro 4, Molecular Devices, San Francisco, CA, United States). Double labeling of LC3 (green) and mRFP (red) immunofluorescence corresponds to changes in autophagic flux. When autophagy and lysosome fusion occur, LC3-GFP fluorescence is quenched, and only red fluorescence can be detected. After merging the red and green fluorescence images, yellow spots in the cell image symbolize autophagosomes.

### Drug Treatment

CAL-101 is a potent and highly selective inhibitor of PI3K, p-AKT, and m-TOR concurrently and has been approved by the Food and Drug Administration (FDA) for the clinical treatment of certain hematological malignancies in 2014. CAL-101 was dissolved in dimethyl sulfoxide (DMSO) and stored at −20°C as a stock solution (10 mM). CNE1R cells were treated with 5 μM CAL-101 in medium for 12 h, and 1% DMSO was used as a control of CAL-101.

### Statistical Analysis

All experiments were repeated at least three times. Data are expressed as mean ± SD and analyzed with the one-way ANOVA method using SPSS17.0 software (SPSS Inc. Chicago, IL, United States). *P* < 0.05 was considered a significant difference between the indicated groups.

## Results

### High ANXA6 Level Is Closely Associated With the Radioresistance of NPC

**FIGURE 1:**
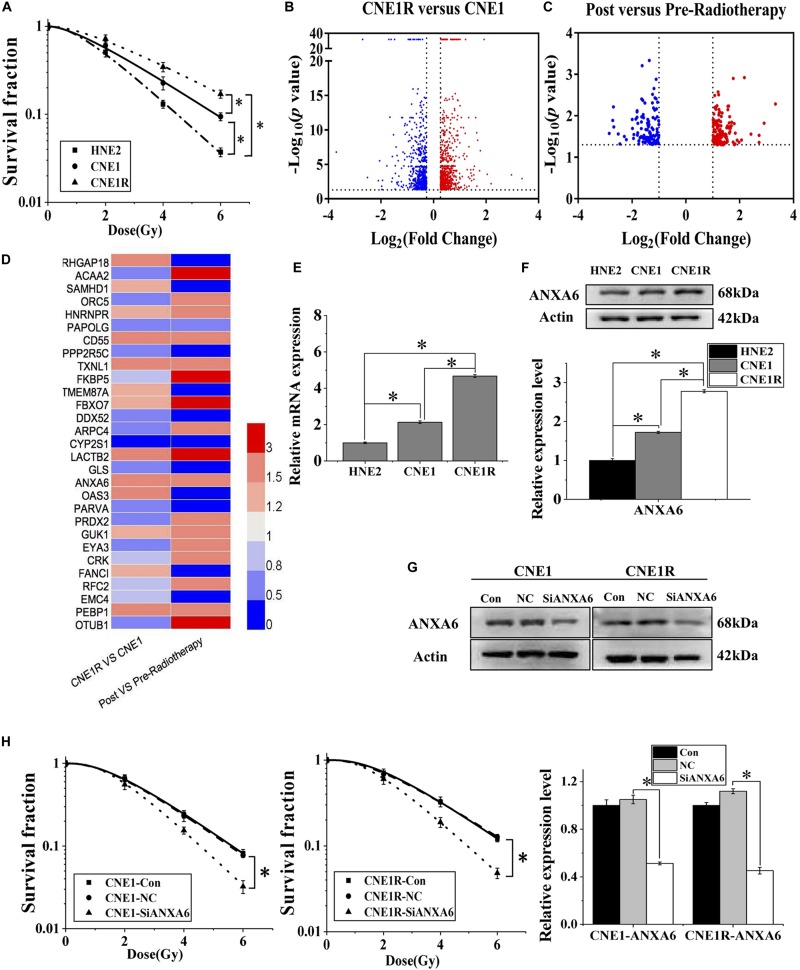
High expression of ANXA6 predicts radioresistance of NPC. **(A)** Survival fractions of HNE2, CNE1, and CNE1R cells after irradiation. **(B)** Volcano plot of differentially expressed genes between CNE1R and CNE1 cells. **(C)** Volcano plot of differentially expressed genes in the tumor tissue of NPC radioresistant patients (*n* = 3) before and after radiotherapy. **(D)** Heat map of the expression levels of 29 differential genes between the above volcano plots, analyzed by Heml (software for drawing volcano maps). **(E)** *ANXA6* mRNA expression levels in HNE2, CNE1, and CNE1R cells. **(F)** Western blot assay of ANXA6 protein in HNE2, CNE1, and CNE1R cells. **(G)** Efficiency of siANXA6 transfection in CNE1 and CNE1R cells. **(H)** Dose responses of survival factions of CNE1 and CNE1R cells after siANXA6 transfection. * P < 0.05 between indicated groups.

Figure 1A illustrates that the two commonly used NPC cell lines, CNE1 and HNE2, had different radiosensitivities and that CNE1 cells were more resistant to radiation. By irradiating CNE1 cells with fractionated doses up to 60 Gy in total, we generated a highly radioresistant cell line named CNE1R. The radioresistance of CNE1R cells was confirmed by the colony-formation assay (Figure 1A).

The proteins from CNE1 cells and its radioresistant counterpart CNE1R were collected and subjected to TMT quantitative proteomic analysis in order to determine the differential proteins between radioresistant cells and their parents. These differentially expressed proteins are illustrated on a volcano plot in Figure 1B. In total, 1,295 differential proteins were identified, among which 658 proteins were upregulated, while 637 were downregulated (Supplementary Table S2, fold change ≥ 1.2, *p-*value < 0.05). Meanwhile, Figure 1C illustrates the gene chip assay results regarding the distribution of differentially expressed genes from three NPC radioresistant patients before and after radiotherapy. Following the calculation criteria (fold change ≥ 2, *p-*value < 0.05), a total of 292 aberrantly expressed genes were obtained, including 155 upregulated and 137 downregulated genes (Supplementary Table S3). Thus, a total of 29 genes were identified from NPC cells and clinical specimens and are visualized by heat map in Figure 1D, including eight co-upregulated genes and six co-downregulated genes. We speculated that the high expression of ANXA6 among them was probably an important prognostic marker of NPC radioresistance because the elevation of ANXA6 has been previously reported to be an independent risk factor for poor prognosis of many neoplasms, such as cervix carcinogenesis, pancreatic cancer, ovarian carcinoma, and thyroid cancer (Lomnytska et al., 2011; O’Sullivan et al., 2017; Lee et al., 2018; Noreen et al., 2019).

Next, we explored the contribution of ANXA6 to the radioresistance of NPC *in vitro*. It was observed that the expression levels of ANXA6 mRNA and protein in three NPC cell lines (HNE2, CNE1, and CNE1R) were positively related to cell radioresistance (Figures 1E,F). To further demonstrate whether *ANXA6* has an essential role in the radioresistance of NPC, the expression of *ANXA6* in CNE1 and CNE1R cells was effectively silenced by siANXA6 (Figure 1G). It was found that transfection of cells with siANXA6 significantly sensitized NPC cells to irradiation and reduced cell survival (Figure 1H).

### Autophagy Contributes to the Radioresistance of NPC Cells

**FIGURE 2:**
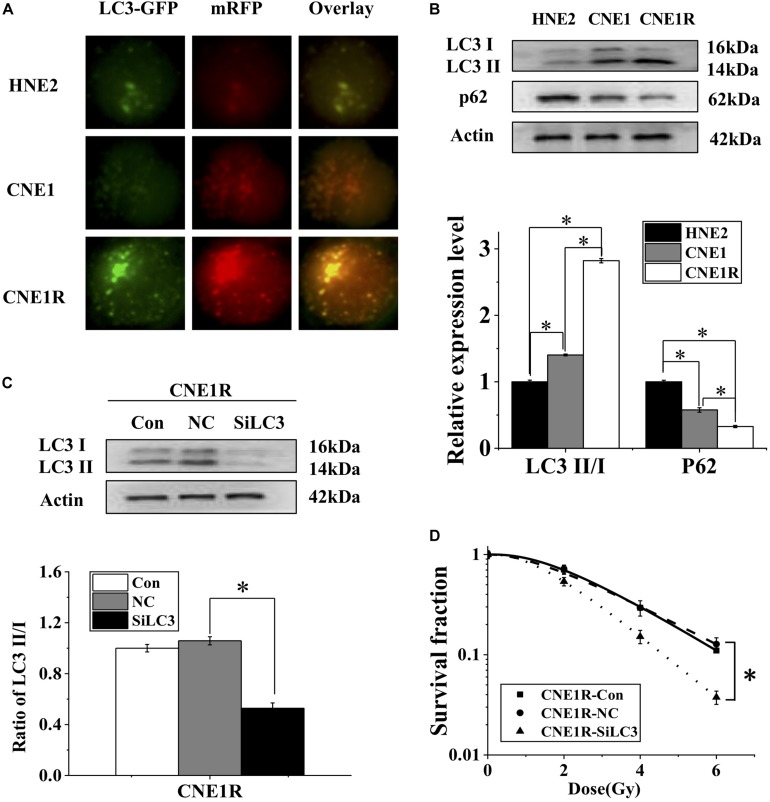
Autophagy contributes to the radioresistance of NPC cells. **(A)** Fluorescence images of HNE2, CNE1, and CNE1R cells transfected with *mRFP-GFP-LC3* (x 40). **(B)** Western blot assay of P62 and LC3 proteins in HNE2, CNE1, and CNE1R cells. **(C)** Efficiency of siLC3 transfection in CNE1R cells. **(D)** Dose responses of survival factions of CNE1R cells before and after siLC3 transfection. *P < 0.05 between indicated groups.

Increasing evidence shows that induction of autophagy contributes to the resistance of anticancer treatments. To determine whether autophagy is involved in the radioresistance of NPC cells, we transfected NPC cells with Ad-*mRFP-GFP-LC3* to label autophagosomes. It was found that the number of LC3 dots increased, in ascending order, in HNE2, CNE1, and CNE1R cells (Figure 2A). Consistently, the ratio of LC3II/LC3I (an autophagic marker) increased, and the autophagy substrate protein p62 decreased in HNE2, CNE1, and CNE1R cells step by step (Figure 2B). To further assess the regulatory effect of autophagy on NPC cells, we transfected cells with siLC3 to block autophagy and then examined whether autophagy could impact the radioresistance of NPC cells. It was found that, when the expressions of LC3I and LC3II were weakened by siLC3, the survival of CNE1R cells was effectively decreased after irradiation (Figures 2C,D). These results suggested that autophagy promoted the radioresistance of NPC cells.

### ANXA6 Regulates Autophagy Induction

**FIGURE 3:**
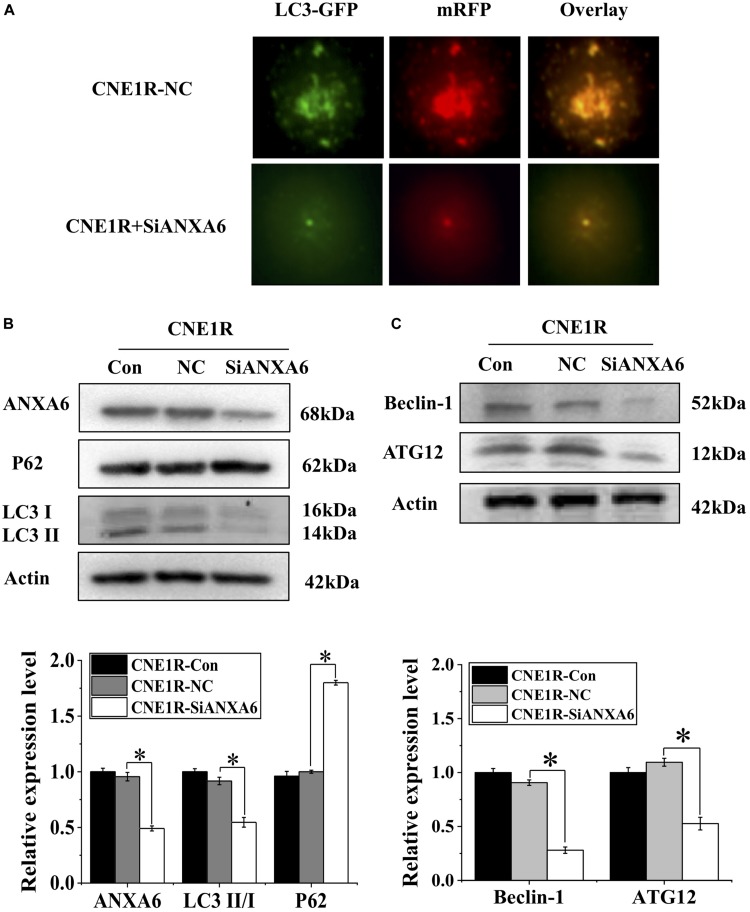
Transfection of siANXA6 inhibits autophagy incidence. **(A)** Fluorescence images of CNE1R cells co-transfected with siANXA6 and mRFP-GFP-LC3 (x40). **(B)** Western blot assay of ANXA6, P62, and LC3 proteins in CNE1R cells. **(C)** Western blot assay of ATG12 and Beclin-1 proteins in CNE1R cells. *P < 0.05 between indicated groups.

To determine whether ANXA6 contributes to autophagy-regulated radioresistance of NPC cells, we transfected radioresistant CNE1R cells with siANXA6 and Ad-*mRFP-GFP-LC3*. As shown in Figure 3A, the number of autophagic LC3 spots in the siANXA6 group decreased sharply in comparison with siRNA negative control. Meanwhile, suppression of ANXA6 decreased the ratio of LC3II/I and thus, in turn, increased p62 expression (Figure 3B). In addition, the expressions of other autophagy-related proteins of Beclin-1 and Autophagy Related 12 (ATG12) also decreased in CNE1R cells after siANXA6 transfection (Figure 3C). Therefore, the silence of ANXA6 significantly inhibited the occurrence of intact autophagic flux.

### ANXA6 Regulates the PI3K/AKT/mTOR Pathway

**FIGURE 4:**
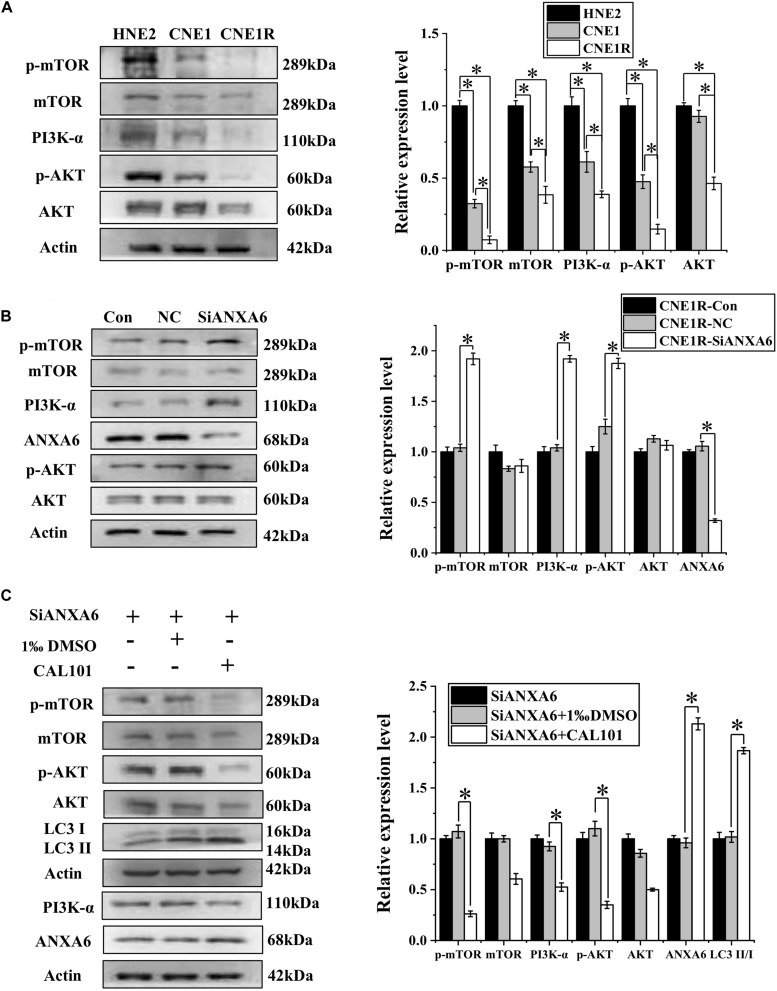
ANXA6 contributes to autophagy incidence via inhibition of the PI3K/AKT/mTOR pathway. **(A)** Western blot assay of PI3K-α, AKT, p-AKT, mTOR, and p-mTOR proteins in HNE2, CNE1, and CNE1R cells. **(B)** Western blot assay of PI3K-α, AKT, p-AKT, mTOR, and p-mTOR proteins in CNE1R cells with or without siANXA6 transfection. **(C)** Western blot assay of PI3K-α, AKT, p-AKT, mTOR, p-mTOR, and ANXA6 proteins in siANXA6-transfected CNE1R cells treated with CAL101 or its control (1‰ DMSO). *P < 0.05 between indicated groups.

To determine the relationship of the PI3K/AKT/mTOR signaling pathway with radioresistance, the expressions of these proteins in NPC cells with different radiosensitivities were measured. Figure 4A illustrates that the expression levels of PI3K-α, p-AKT, and p-mTOR decreased along with the radiosensitivity of HNE2, CNE1, and CNE1R cells, in contrast to the LC3II/I ratio in these cells. When the most radioresistant cells of CNE1R were transfected with siANXA6, the levels of PI3K-α, p-AKT, and p-mTOR were obviously increased in comparison with those in the siRNA control cells (Figure 4B). Therefore, knockdown of ANXA6 activates the PI3K/AKT/mTOR signaling pathway.

Given our observation of the relationship among ANXA6, PI3K/AKT/mTOR, and autophagy, CNE1R cells transferred with siANXA6 were further treated with CAL101, a specific inhibitor of the PI3K/AKT/mTOR pathway. It was found that CAL101 effectively decreased the expressions of PI3K-α, p-AKT, and p-mTOR. Furthermore, when cells were co-treated with siANXA6 and CAL101, both ANXA6 and LC3II/I ratio were extensively increased in comparison with the siANXA6 alone group, suggesting that the decrease of autophagy induced by siANXA6 could be reversed by blocking the PI3K/AKT/mTOR pathway (Figure 4C). Taken together, these results indicate that ANXA6 upregulated the radioresistance of NPC cells by promoting autophagy through the inhibition of the PI3K/AKT/mTOR signaling pathway.

## Discussion

Due to the anatomical location and radiosensitivity of NPC, radiotherapy is the primary treatment method of this type of carcinoma, different from most other malignant tumors (Chua et al., 2016; Sun et al., 2016). Current advances in comprehensive population screening and effective drugs have significantly reduced nasopharyngeal cancer mortality, but recurrence and resistance after radiotherapy remain a problem in NPC, and the molecular mechanism of NPC radioresistance is still unresolved.

To further verify the relationship between ANXA6 and radioresistance, we performed a comprehensive analysis of data from the Gene Expression Profiling Interactive Analysis (GEPIA) database and The Cancer Genome Atlas (TCGA) database and found that 10 kinds of tumors overexpress ANXA6 in comparison to their normal tissues (Supplementary Figure S1). Besides the four cancers mentioned in the above results section, patients harboring a high expression of ANXA6 also possess a poor prognosis in bladder urothelial carcinoma (BLCA), kidney renal papillary cell carcinoma (KIRP), lung squamous cell carcinoma (LUSC), and mesothelioma (MESO) (Supplementary Figure S2), denoting that the overexpression of ANXA6 might serve as a crucial contributor to develop anti-tumor therapy strategy, especially for radioresistance. Though recent studies have revealed that the expression of ANXA6 is bound up with chemoresistance and poor prognosis of malignant tumors (Lomnytska et al., 2011; Keklikoglou et al., 2019), its role in radioresistance in various kinds of tumors has not yet been reported. In this study, we innovatively observed a positive relationship between ANXA6 expression and the radioresistance of NPC in both cell lines and clinical patients.

It was known that ANXA6 can enhance autophagy in rat liver hepatocytes and trigger endocytic transport and lysosome fusion by inducing re-arrangements of specific lipids and calcium channel transfer (Enrich et al., 2017). But the role of autophagy in radioresistance is still controversial. Wang et al. (2013) hold the view that autophagy promotes radioresistance in pancreatic cancer cells, while Djavid et al. (2017) concluded that the mobilization of autophagy could cause cell death through lysosomal activation, thus causing an enhancement of radiosensitivity in human cervical cancer cells. Our study demonstrated that autophagy inhibition increased the radiosensitivity of NPC cells and that there was a positive correlation between autophagy and radioresistance. Apart from the physiological regulation of calcium channels and re-arrangements of specific lipids, we found that ANXA6 could strengthen autophagy by blocking the PI3K/AKT/mTOR pathway. Meanwhile, siANXA6-suppressed autophagy could be reversed by a PI3K/AKT/mTOR inhibitor of CAL101. Taking these results together, we suggest that PI3K/AKT/mTOR complements the mechanism of the incidence of autophagy regulated by ANXA6.

The PI3K/AKT/mTOR pathway is one of the most frequently activated signaling pathways in cancers and is responsible for tumor development, cellular metastasis, and proliferation (Polivka and Janku, 2014; Zhang et al., 2017; Chamcheu et al., 2019). In particular, the PI3K/AKT/mTOR pathway has attracted extensive attention as the modulator of autophagy (Xu et al., 2020). The central checkpoint for the negative regulation of autophagy is mTOR, and anti-tumor drugs stimulate autophagy by attenuating the PI3K/AKT/mTOR pathway (Janku et al., 2011). Recent advances in radiotherapy also indicate that triggering of the PI3K/AKT/mTOR pathway is closely relevant to radioresistance, which is a major challenge for current radiation treatment in prostate cancer (CaP) and other cancers (Heavey et al., 2014; Chang et al., 2015). However, in our study, the PI3K/AKT/mTOR pathway was considered as a negative regulator for tumor progression and radiation resistance. According to many studies, the inhibition of the PI3K/AKT/mTOR pathway may contribute to weakened proliferation (Feng and Qiu, 2018) and prolonged cell cycle (He et al., 2018), which may provide much time for autophagy to phagocytize damaged organelles so as to maintain the stability of the intracellular environment, thus promoting cell survival and radioresistance after irradiation. Besides, it has been reported that targeting PI3K/AKT/mTOR-mediated autophagy strongly enhances the chemosensitivity of tumor cells. The overexpression of miR-142-3p attenuated autophagy by regulating the PI3K/AKT/mTOR pathway and enhanced the chemosensitivity of non-small cell lung cancers (Chen et al., 2017). For hepatocarcinoma, erlotinib induces autophagy through blocking the PI3K/AKT/mTOR pathway to enhance tumor resistance (Li et al., 2019). Another study reported that ZD6474, a small-molecule inhibitor that suppresses the activities of epidermal growth factor receptor, vascular endothelial growth factor receptor, and tyrosine kinases receptor, can activate autophagy depending on attenuation of the PI3K/AKT/mTOR signaling pathway to protect glioblastoma cells (Shen et al., 2013). These studies have demonstrated that the overexpression of the PI3K/AKT/mTOR pathway is beneficial for subduing autophagy and reducing tumor resistance.

Mechanistically, the phosphorylation of EGFR can stimulate several downstream signaling pathways, including MAPK/MEK/ERK and PI3K/AKT, that are involved in a variety of mitogenic, metastatic, and other tumor-promoting cellular activities (Wells, 1999; Roberts and Der, 2007). It was reported that an elevated ANXA6 level could inhibit the phosphorylation of EGFR by promoting protein kinase Cα (PKCα) and restrained EGFR signaling (Koese et al., 2013). Thus, we infer that the negative effect of ANXA6 on the PI3K/AKT/mTOR pathway may result from its inhibition of EGFR phosphorylation, though this needs further verification.

**FIGURE 5:**
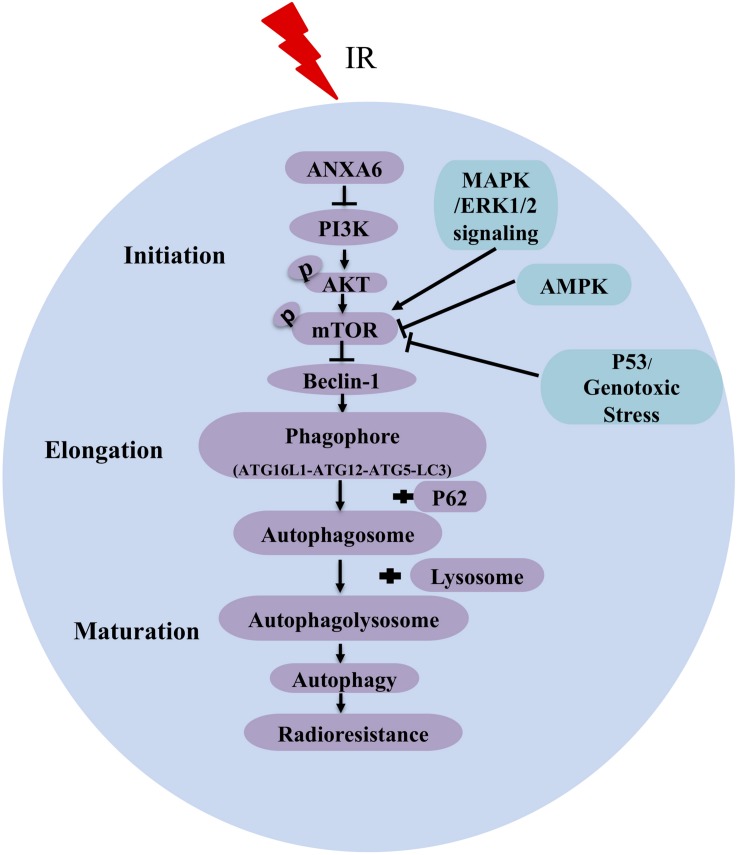
A pattern diagram shows that ANXA6 regulates autophagy via inhibiting the PI3K/AKT/mTOR pathway to induce radioresistance of NPC. Irradiation (IR) increases the expression of ANXA6 in NPC cell lines and patient tumor tissues, which, in turn, directly inhibits the expression of PI3K and further reduces the phosphorylation of AKT and mTOR. The reduced p-mTOR increases the expression of Beclin-1 (a key activator of autophagy) and advances the formation of phagophore consisting of ATG16L1 (autophagy related 16-like 1), ATG12, ATG5 (autophagy related protein 5), and LC3 protein. Additionally, P62 binds to autophagosomal membrane protein LC3 and delivers itself to autophagosome, which ultimately leads to a decline of P62. Finally, autophagolysosome formed by the fusion of autophagosome and lysosome can phagocytize injured organelles to maintain/restore metabolic homeostasis, contributing to the radioresistance of NPC. Arrows represent promotion events, and blunt arrows indicate suppression events.

In summary, our results demonstrate that ANXA6-regulated autophagy via the PI3K/AKT/mTOR pathway makes a major contribution to the radioresistance of NPC (Figure 5). It is worth mentioning that this study has revealed the connection between ANXA6 and radiosensitivity for the first time and further implies that ANXA6 may be applied as a new biomarker for the diagnosis and prognosis of NPC radiotherapy. In addition, it will be of great benefit to expand the research on ANXA6 to head and neck neoplasms as a potential therapeutic target for radiosensitization in the future.

## Data Availability Statement

The raw data supporting the conclusions of this article will be made available by the authors, without undue reservation, to any qualified researcher.

## Ethics Statement

The studies involving human participants were reviewed and approved by the Nanfang Hospital of Southern Medical University Institutional Board. The patients/participants provided their written informed consent to participate in this study.

## Author Contributions

CS, QC, WZ, and LZ made contributions to the conception and design of the study. DY, CW, YS, SH, and HL performed acquisition and analysis of data. YB and JZ performed the statistical analysis. QC wrote the first draft of the manuscript. CS, JG, and YP revised the manuscript.

## Conflict of Interest

The authors declare that the research was conducted in the absence of any commercial or financial relationships that could be construed as a potential conflict of interest.

## References

[B1] Cai C. Min S. Yan B. Liu W. Yang X. Li L. (2019). MiR-27a promotes the autophagy and apoptosis of IL-1beta treated-articular chondrocytes in osteoarthritis through PI3K/AKT/mTOR signaling. *Aging* 11 6371–6384. 10.18632/aging.102194 31460867 PMC6738432

[B2] Chaachouay H. Fehrenbacher B. Toulany M. Schaller M. Multhoff G. Rodemann H. P. (2015). AMPK-independent autophagy promotes radioresistance of human tumor cells under clinical relevant hypoxia in vitro. *Radiother. Oncol.* 116 409–416. 10.1016/j.radonc.2015.08.012 26318663

[B3] Chaachouay H. Ohneseit P. Toulany M. Kehlbach R. Multhoff G. Rodemann H. P. (2011). Autophagy contributes to resistance of tumor cells to ionizing radiation. *Radiother. Oncol.* 99 287–292. 10.1016/j.radonc.2011.06.002 21722986

[B4] Chamcheu J. C. Roy T. Uddin M. B. Banang-Mbeumi S. Chamcheu R. N. Walker A. L. (2019). Role and therapeutic targeting of the pi3k/akt/mtor signaling pathway in skin cancer: a review of current status and future trends on natural and synthetic agents therapy. *Cells Basel* 8:803. 10.3390/cells8080803 31370278 PMC6721560

[B5] Chang L. Graham P. H. Ni J. Hao J. Bucci J. Cozzi P. J. (2015). Targeting PI3K/Akt/mTOR signaling pathway in the treatment of prostate cancer radioresistance. *Crit. Rev. Oncol. Hematol.* 96 507–517. 10.1016/j.critrevonc.2015.07.005 26253360

[B6] Chen W. Hu G. H. (2015). Biomarkers for enhancing the radiosensitivity of nasopharyngeal carcinoma. *Cancer Biol. Med.* 12 23–32. 10.7497/j.issn.2095-3941.2014.0015 25859408 PMC4383846

[B7] Chen Y. Zhou X. Qiao J. Bao A. (2017). MiR-142-3p overexpression increases chemo-sensitivity of NSCLC by Inhibiting HMGB1-mediated autophagy. *Cell Physiol. Biochem.* 41 1370–1382. 10.1159/000467896 28427045

[B8] Chen Y. P. Chan A. T. C. Le Q. T. Blanchard P. Sun Y. Ma J. (2019). Nasopharyngeal carcinoma. *Lancet* 394 64–80. 10.1016/S0140-6736(19)30956-0 31178151

[B9] Choi A. M. Ryter S. W. Levine B. (2013). Autophagy in human health and disease. *N. Engl. J. Med.* 368 651–662. 10.1056/NEJMra1205406 23406030

[B10] Chua M. L. K. Wee J. T. S. Hui E. P. Chan A. T. C. (2016). Nasopharyngeal carcinoma. *Lancet* 387 1012–1024. 10.1016/S0140-6736(15)00055-0 26321262

[B11] Djavid G. E. Bigdeli B. Goliaei B. Nikoofar A. Hamblin M. R. (2017). Photobiomodulation leads to enhanced radiosensitivity through induction of apoptosis and autophagy in human cervical cancer cells. *J. Biophotonics* 10 1732–1742. 10.1002/jbio.201700004 28464474 PMC5668202

[B12] Enrich C. Rentero C. de Muga S. V. Reverter M. Mulay V. Wood P. (2011). Annexin A6-Linking Ca(2+) signaling with cholesterol transport. *Biochim. Biophys. Acta* 1813 935–947. 10.1016/j.bbamcr.2010.09.015 20888375

[B13] Enrich C. Rentero C. Grewal T. (2017). Annexin A6 in the liver: from the endocytic compartment to cellular physiology. *Biochim. Biophys. Acta Mol. Cell Res.* 1864 933–946. 10.1016/j.bbamcr.2016.10.017 27984093

[B14] Feng F. B. Qiu H. Y. (2018). Effects of Artesunate on chondrocyte proliferation, apoptosis and autophagy through the PI3K/AKT/mTOR signaling pathway in rat models with rheumatoid arthritis. *Biomed. Pharmacother.* 102 1209–1220. 10.1016/j.biopha.2018.03.142 29710540

[B15] Ferlay J. Soerjomataram I. Dikshit R. Eser S. Mathers C. Rebelo M. (2015). Cancer incidence and mortality worldwide: sources, methods and major patterns in GLOBOCAN 2012. *Int. J. Cancer* 136 E359–E386. 10.1002/ijc.29210 25220842

[B16] Grewal T. Koese M. Rentero C. Enrich C. (2010). Annexin A6-regulator of the EGFR/Ras signalling pathway and cholesterol homeostasis. *Int. J. Biochem. Cell Biol.* 42 580–584. 10.1016/j.biocel.2009.12.020 20044025

[B17] He D. Sun X. Yang H. Li X. Yang D. (2018). TOFA induces cell cycle arrest and apoptosis in ACHN and 786-O cells through inhibiting PI3K/Akt/mTOR pathway. *J. Cancer* 9 2734–2742. 10.7150/jca.26374 30087714 PMC6072807

[B18] Heavey S. O’Byrne K. J. Gately K. (2014). Strategies for co-targeting the PI3K/AKT/mTOR pathway in NSCLC. *Cancer Treat. Rev.* 40 445–456. 10.1016/j.ctrv.2013.08.006 24055012

[B19] Janku F. McConkey D. J. Hong D. S. Kurzrock R. (2011). Autophagy as a target for anticancer therapy. *Nat. Rev. Clin. Oncol.* 8 528–539. 10.1038/nrclinonc.2011.71 21587219

[B20] Katheder N. S. Rusten T. E. (2017). Microenvironment and tumors-a nurturing relationship. *Autophagy* 13 1241–1243. 10.1080/15548627.2017.1310361 28632995 PMC5529065

[B21] Keklikoglou I. Cianciaruso C. Guc E. Squadrito M. L. Spring L. M. Tazzyman S. (2019). Chemotherapy elicits pro-metastatic extracellular vesicles in breast cancer models. *Nat. Cell Biol.* 21 190–202. 10.1038/s41556-018-0256-3 30598531 PMC6525097

[B22] Koese M. Rentero C. Kota B. P. Hoque M. Cairns R. Wood P. (2013). Annexin A6 is a scaffold for PKCalpha to promote EGFR inactivation. *Oncogene* 32 2858–2872. 10.1038/onc.2012.303 22797061

[B23] Koukourakis M. I. Mitrakas A. G. Giatromanolaki A. (2016). Therapeutic interactions of autophagy with radiation and temozolomide in glioblastoma: evidence and issues to resolve. *Br. J. Cancer* 114 485–496. 10.1038/bjc.2016.19 26889975 PMC4782209

[B24] Lee A. W. Sze W. M. Au J. S. Leung S. F. Leung T. W. Chua D. T. (2005). Treatment results for nasopharyngeal carcinoma in the modern era: the Hong Kong experience. *Int. J. Radiat. Oncol. Biol. Phys.* 61 1107–1116. 10.1016/j.ijrobp.2004.07.702 15752890

[B25] Lee H. S. Kang Y. Tae K. Bae G. U. Park J. Y. Cho Y. H. (2018). Proteomic biomarkers for bisphenol a-early exposure and women’s thyroid cancer. *Cancer Res. Treat.* 50 111–117. 10.4143/crt.2017.001 28279065 PMC5784619

[B26] Li W. Y. Li Q. Jing L. Wu T. Han L. L. Wang Y. (2019). P57-mediated autophagy promotes the efficacy of EGFR inhibitors in hepatocellular carcinoma. *Liver Int.* 39 147–157. 10.1111/liv.13957 30178471

[B27] Li Z. Q. Xia Y. F. Liu Q. Yi W. Liu X. F. Han F. (2006). Radiotherapy-related typing in 842 patients in canton with nasopharyngeal carcinoma. *Int. J. Radiat. Oncol. Biol. Phys.* 66 1011–1016. 10.1016/j.ijrobp.2006.06.028 16997506

[B28] Liu S. C. Tsang N. M. Chiang W. C. Chang K. P. Hsueh C. Liang Y. (2013). Leukemia inhibitory factor promotes nasopharyngeal carcinoma progression and radioresistance. *J. Clin. Invest.* 123 5269–5283. 10.1172/JCI63428 24270418 PMC3859424

[B29] Lomnytska M. I. Becker S. Bodin I. Olsson A. Hellman K. Hellstrom A. C. (2011). Differential expression of ANXA6, HSP27, PRDX2, NCF2, and TPM4 during uterine cervix carcinogenesis: diagnostic and prognostic value. *Br. J. Cancer* 104 110–119. 10.1038/sj.bjc.6605992 21119665 PMC3039821

[B30] Noreen S. Gardner Q. A. Fatima I. Sadaf S. Akhtar M. W. (2019). Upregulated expression of calcium-dependent annexin a6: a potential biomarker of ovarian carcinoma. *Proteomics Clin. Appl.* 14:e1900078. 10.1002/prca.201900078 31747122

[B31] O’Sullivan D. Dowling P. Joyce H. McAuley E. McCann A. Henry M. (2017). A novel inhibitory anti-invasive MAb isolated using phenotypic screening highlights AnxA6 as a functionally relevant target protein in pancreatic cancer. *Br. J. Cancer* 117 1326–1335. 10.1038/bjc.2017.306 28881357 PMC5672937

[B32] Polivka J. Jr. Janku F. (2014). Molecular targets for cancer therapy in the PI3K/AKT/mTOR pathway. *Pharmacol. Ther.* 142 164–175. 10.1016/j.pharmthera.2013.12.004 24333502

[B33] Roberts P. J. Der C. J. (2007). Targeting the Raf-MEK-ERK mitogen-activated protein kinase cascade for the treatment of cancer. *Oncogene* 26 3291–3310. 10.1038/sj.onc.1210422 17496923

[B34] Shen J. Zheng H. Ruan J. Fang W. Li A. Tian G. (2013). Autophagy inhibition induces enhanced proapoptotic effects of ZD6474 in glioblastoma. *Br. J. Cancer* 109 164–171. 10.1038/bjc.2013.306 23799852 PMC3708568

[B35] Sun Y. Li W. F. Chen N. Y. Zhang N. Hu G. Q. Xie F. Y. (2016). Induction chemotherapy plus concurrent chemoradiotherapy versus concurrent chemoradiotherapy alone in locoregionally advanced nasopharyngeal carcinoma: a phase 3, multicentre, randomised controlled trial. *Lancet Oncol.* 17 1509–1520. 10.1016/S1470-2045(16)30410-7 27686945

[B36] Varshney P. Saini N. (2018). PI3K/AKT/mTOR activation and autophagy inhibition plays a key role in increased cholesterol during IL-17A mediated inflammatory response in psoriasis. *Biochim. Biophys. Acta Mol. Basis Dis.* 1864 1795–1803. 10.1016/j.bbadis.2018.02.003 29432814

[B37] Wang P. Zhang J. Zhang L. Zhu Z. Fan J. Chen L. (2013). MicroRNA 23b regulates autophagy associated with radioresistance of pancreatic cancer cells. *Gastroenterology* 145 e1112. 10.1053/j.gastro.2013.07.048 23916944

[B38] Wang X. Fu Y. F. Liu X. Feng G. Xiong D. Mu G. F. (2018). ROS promote Ox-LDL-induced platelet activation by up-regulating autophagy through the inhibition of the PI3K/AKT/mTOR pathway. *Cell Physiol. Biochem.* 50 1779–1793. 10.1159/000494795 30384368

[B39] Wells A. (1999). EGF receptor. *Int. J. Biochem. Cell Biol.* 31 637–643. 10.1016/s1357-2725(99)00015-1 10404636

[B40] Xu Z. Han X. Ou D. Liu T. Li Z. Jiang G. (2020). Targeting PI3K/AKT/mTOR-mediated autophagy for tumor therapy. *Appl. Microbiol. Biotechnol.* 104 575–587. 10.1007/s00253-019-10257-8 31832711

[B41] Yao K. C. Komata T. Kondo Y. Kanzawa T. Kondo S. Germano I. M. (2003). Molecular response of human glioblastoma multiforme cells to ionizing radiation: cell cycle arrest, modulation of the expression of cyclin-dependent kinase inhibitors, and autophagy. *J. Neurosurg.* 98 378–384. 10.3171/jns.2003.98.2.0378 12593626

[B42] Zhang M. Liu S. Chua M. S. Li H. Luo D. Wang S. (2019). SOCS5 inhibition induces autophagy to impair metastasis in hepatocellular carcinoma cells via the PI3K/Akt/mTOR pathway. *Cell Death Dis.* 10 799 10.1038/s41419-019-1856-y 31641102 PMC6805927

[B43] Zhang Y. Kwok-Shing, Ng P. Kucherlapati M. Chen F. Liu Y. (2017). A pan-cancer proteogenomic atlas of pi3k/akt/mtor pathway alterations. *Cancer Cell* 31 820.e3–832.e3. 10.1016/j.ccell.2017.04.013 28528867 PMC5502825

