# Supplementary material for: ANXA6 Contributes to Radioresistance by Promoting Autophagy via Inhibiting the PI3K/AKT/mTOR Signaling Pathway in Nasopharyngeal Carcinoma
Source: Front Cell Dev Biol. 2020 Apr 16;8:232. doi: 10.3389/fcell.2020.00232 (PMC7176914; doi:10.3389/fcell.2020.00232)
Supplement: Supplementary file 1 [file Data_Sheet_1.PDF]

Supplementary figures

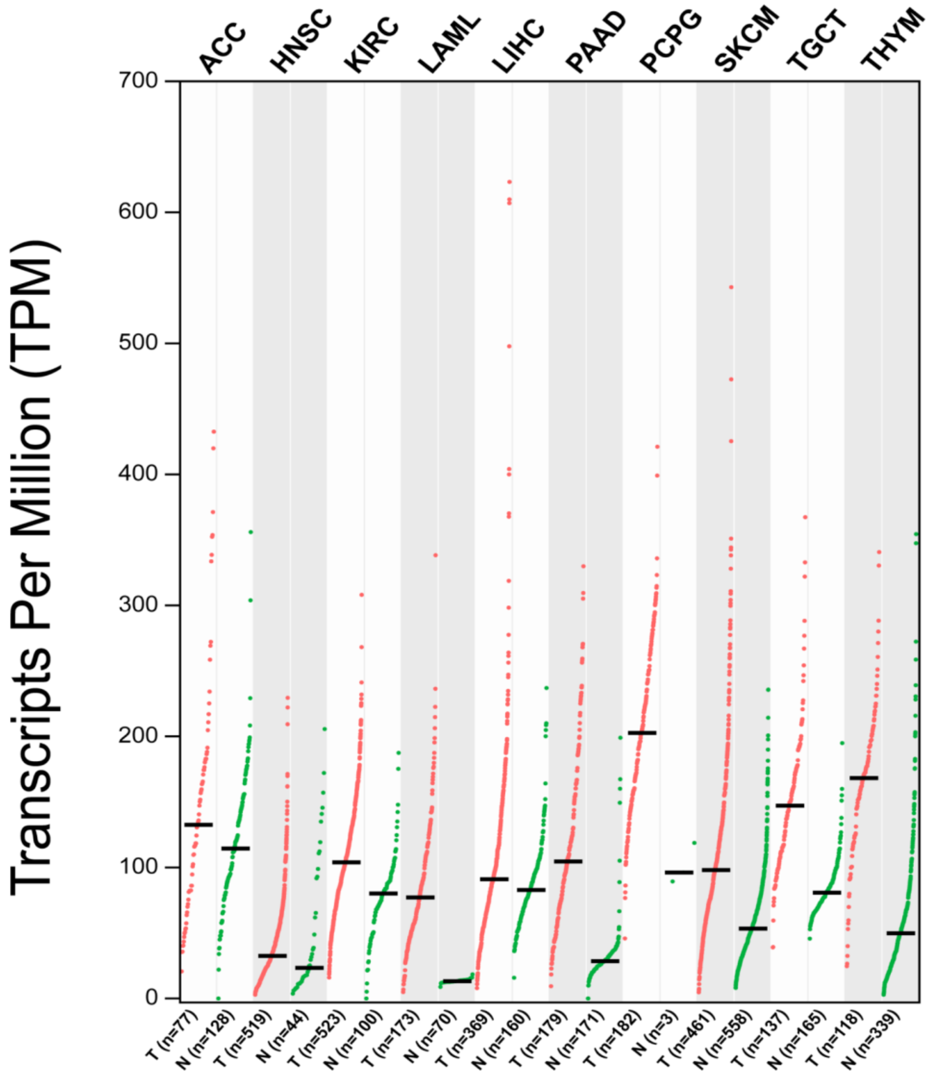

FIGURE S1: ANXA6 is overexpressed in 10 types of tumors, which is analyzed from the data from GEPIA and TCGA database.

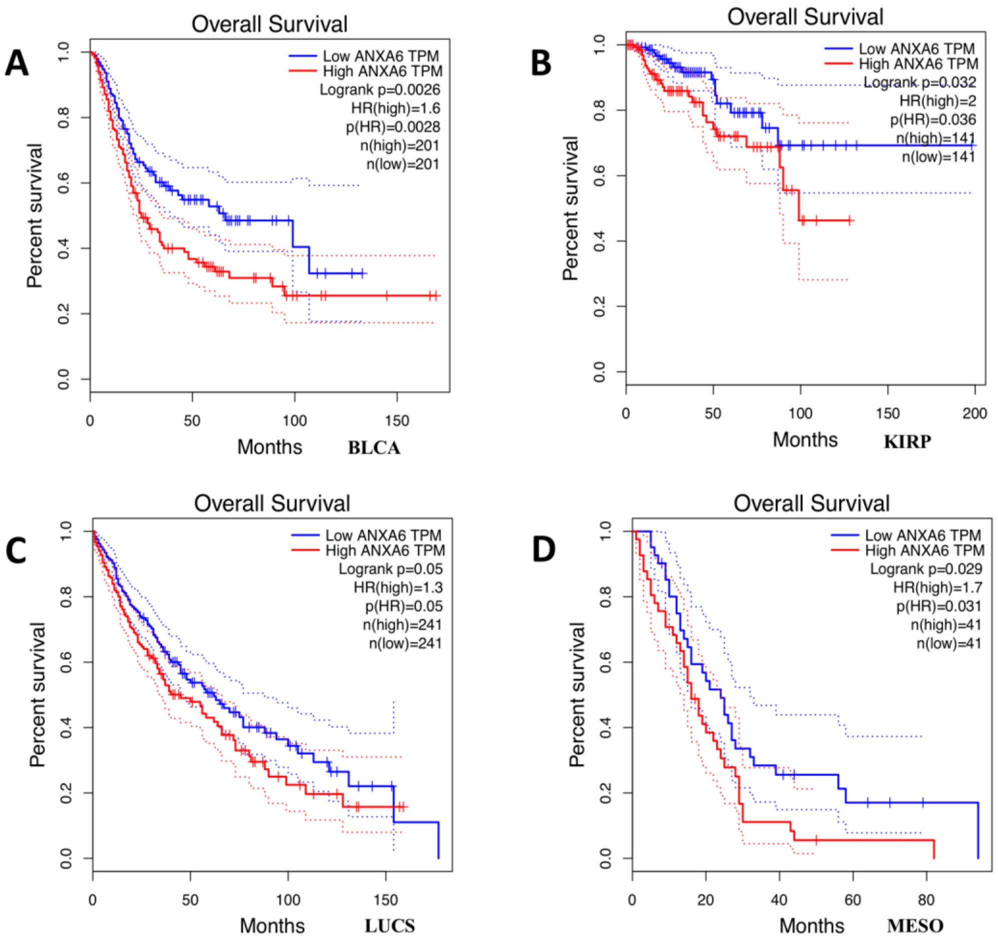

FIGURE S2: ANXA6 overexpression predicts a poor prognosis in neoplastic disease based on GEPIA and TCGA database. **A–D:** Patients with high expression of ANXA6 possesses a poor prognosis in bladder urothelial carcinoma (BLCA), kidney renal papillary cell carcinoma (KIRP), lung squamous cell carcinoma (LUSC), and mesothelioma (MESO).
